# Computational Study of Hemodynamic Changes Induced by Overlapping and Compacting of Stents and Flow Diverter in Cerebral Aneurysms

**DOI:** 10.3389/fneur.2021.705841

**Published:** 2021-08-02

**Authors:** Sunghan Kim, Hyeondong Yang, Ineui Hong, Je Hoon Oh, Yong Bae Kim

**Affiliations:** ^1^Department of Neurosurgery, Bucheon St. Mary's Hospital, College of Medicine, The Catholic University of Korea, Seoul, South Korea; ^2^Department of Neurosurgery, Severance Stroke Center, Severance Hospital, Yonsei University College of Medicine, Seoul, South Korea; ^3^Department of Mechanical Engineering and BK21 FOUR ERICA-ACE Center, Hanyang University, Ansan, South Korea

**Keywords:** stent, flow diverter, flow diversion effect, metal coverage rate, overlapping, compaction, computational fluid dynamics

## Abstract

**Purpose:** The flow diversion effect of an intracranial stent is closely related to its metal coverage rate (MCR). In this study, the flow diversion effects of Enterprise and low-profile visualized intraluminal support (LVIS) stents are compared with those of a Pipeline flow diverter, focusing on the MCR change. Moreover, the changes in the flow diversion effect caused by the additional manipulations of overlapping and compaction are verified using computational fluid dynamics (CFD) analysis.

**Methods:** CFD analysis was performed using virtually generated stents mounted in an idealized aneurysm model. First, the flow diversion effects of single Enterprise, LVIS, and Pipeline devices were analyzed. The Enterprise and LVIS were sequentially overlapped and compared with a Pipeline, to evaluate the effect of stent overlapping. The effect of compacting a stent was evaluated by comparing the flow diversion effects of a single and two compacted LVIS with those of two overlapped, uncompacted LVIS and uncompacted and compacted Pipeline. Quantitative analysis was performed to evaluate the hemodynamic parameters of energy loss, average velocity, and inflow rate.

**Results:** Statistically significant correlations were observed between the reduction rates of the hemodynamic parameters and MCR. The single LVIS without compaction induced a reduction in all the hemodynamic parameters comparable to those of the three overlapped Enterprise. Moreover, the two overlapped, uncompacted LVIS showed a flow diversion effect as large as that induced by the single uncompacted Pipeline. Compacted stents induced a better flow diversion effect than uncompacted stents. The single compacted LVIS induced a flow diversion effect similar to that induced by the two uncompacted LVIS or single uncompacted Pipeline.

**Conclusions:** The MCR of a stent correlates with its flow diversion effect. Overlapping and compaction can increase the MCR of an intracranial stent and achieve a flow diversion effect as large as that observed with a flow diverter.

## Introduction

Intracranial, self-expanding stents were originally designed as scaffolding to protect aneurysmal necks against coil protrusion or migration (1, 2). Recently, the flow diversion effect of intracranial stents has received considerable attention (3). This effect describes a phenomenon in which the blood flow into an aneurysmal sac is redirected by a stent implanted in the parent artery (4). The flow diversion effect promotes the potential for postembolization thrombosis, which improves the success rate of aneurysm treatment (5, 6).

Stents currently available on the market have different mechanical properties depending on their design and manufacturing methods (7, 8). The mechanical properties of a stent affect the results of aneurysm treatment, and the metal coverage rate (MCR) of a stent is closely related to the flow diversion effect (9). The MCR indicates the percentage of the aneurysmal neck covered by metal after the application of a stent (10). According to previous studies, the aneurysm occlusion rate correlates positively with the MCR (11, 12). Therefore, achieving a high MCR is a key factor in the success of stent-assisted aneurysm treatment.

Each stent has a constant range of MCR that depends on its mechanical properties. However, a higher MCR can be achieved by using an adjuvant method. Overlapping multiple stents is one of the methods commonly used to increase the MCR. Previous studies have reported that sequentially placing stents across the aneurysm neck can enhance the flow diversion effect (13–15). Another way to increase the MCR is to use the properties of braided stents, whose MCR can be changed by compaction. Unlike a laser-cut stent, a braided stent can produce various mesh densities as the wires of the stent are rearranged according to the device size, vessel diameter, and curvature (16). Compacting a braided stent using the push-pull technique can result in a higher MCR around the aneurysm neck, which can improve the aneurysm occlusion rate (17–19).

In actual aneurysm treatment, stents are overlapped or compacted to induce the flow diversion effect, and sometimes these manipulations are used together. Therefore, a comprehensive understanding of the flow diversion effect induced by overlapping or compacting a stent is necessary. However, no study conducted so far has compared the effect of overlapping and compacting intracranial stents in a single configuration on the flow diversion effect. Although previous studies have demonstrated the flow diversion effect of stents that were separately overlapped or compacted, variations in the configurations used in these studies should be considered while comparing their results (20, 21).

In this study, we compared the flow diversion effects of an Enterprise laser-cut stent (Cerenovus, Raynham, Massachusetts, USA) and a low-profile visualized intraluminal support (LVIS) braided stent (MicroVention, Tustin, California, USA) with that of a Pipeline flow diverter (Medtronic Neurovascular, Irvine, California, USA). The MCR was calculated and compared to evaluate the flow diversion effect of each stent numerically, considering additional manipulations, namely, overlapping and compaction. All the studies were conducted under the same experimental conditions using an idealized aneurysm model to control for variables that could affect the results. Thus, we compared the flow diversion effects of the Enterprise and LVIS stents with that of the Pipeline flow diverter, focusing on the MCR changes. Furthermore, we verified the changes in the flow diversion effect caused by the additional manipulations of overlapping and compaction using computational fluid dynamics (CFD) analysis.

## Materials and Methods

### Aneurysm and Stent Modeling

**Figure 1:**
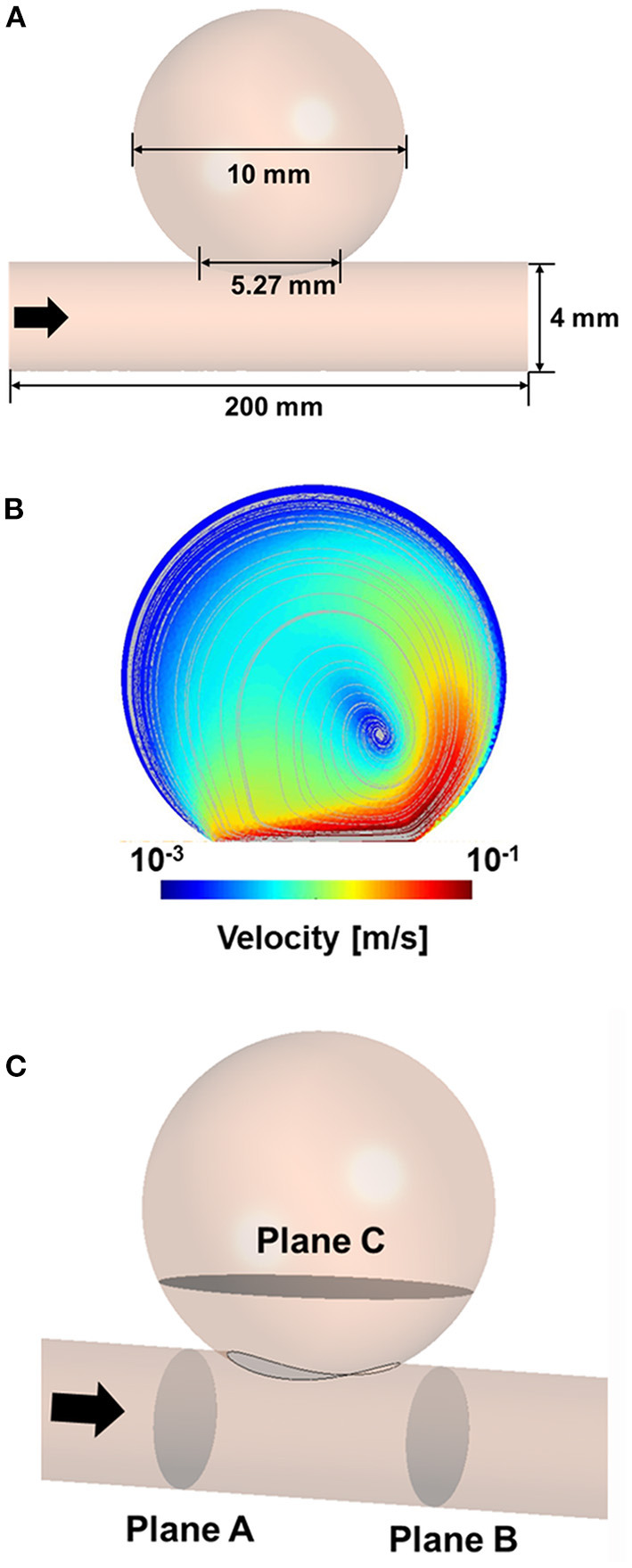
Three-dimensional model of idealized sidewall-type aneurysm for CFD analysis **(A)**. CFD velocity and streamline results for CFD process validation **(B)**. Position of the planes for extracting the hemodynamic parameters **(C)**. CFD, computational fluid dynamics.

An idealized sidewall-type saccular aneurysm model that was minimally affected by lesion geometry was established to compare the characteristics of the stents (Figure 1A). The ideal sidewall aneurysm model had a radius of 5 mm and a neck diameter of 5.27 mm, and the diameter of a parent artery was 4 mm. The sizes of the ideal aneurysm and its parent artery were set by assuming a large aneurysm in the internal carotid artery.

A silicone model of the ideal aneurysm was fabricated, and the real stents were deployed inside the silicone model *in vitro* to examine the configurations of the stents in the aneurysm neck. After installing each real stent inside the silicone model, we captured the images of the stent shapes in the aneurysm neck. Based on the information obtained from the images of deployed stents, we generated virtual stents for subsequent CFD analyses.

CFD analyses were performed using an idealized aneurysm model with virtually created stents placed across the aneurysmal neck. We considered three different kinds of stents: a laser-cut stent (Enterprise, 4.5 × 39 mm), a braided stent (LVIS, 3.5 × 22 mm), and a flow diverter (Pipeline, 4.0 × 35 mm). The size of each stent was set to match the size of the parent artery as much as possible. In the absence of a stent of the same size as the parent artery, an undersized stent was chosen to maximize the MCR (22). This study aims to answer the following questions:

① How large is the flow diversion effect of the Enterprise and LVIS stents compared with that of the Pipeline flow diverter?
② What is the influence of an overlap of the Enterprise or LVIS stents on the flow diversion effect compared with that of a single placement?
③ What is the influence of compaction of an LVIS stent or Pipeline flow diverter on the flow diversion effect compared with that of an uncompacted state?

First, the flow diversion effects of a single Enterprise stent, LVIS stent, and Pipeline flow diverter were analyzed separately. Then, the Enterprise and LVIS stents were sequentially overlapped virtually, and their flow diversion effects were compared with that of the Pipeline flow diverter. The virtual stents were overlapped to have a constant gap between them, to investigate the results of ideal overlapped stents. The CFD results for the LVIS and Pipeline devices were compared with and without compaction to evaluate the flow diversion effect according to stent compaction. The compaction study did not include the Enterprise device because it cannot be compacted owing to its manufacturing method (8). During the compaction study, the maximum compaction rate was achieved by examining the configuration of the real stent mounted in the silicone model aneurysm.

### Validation of the CFD Process

To verify our CFD process, we used the experimental data of Tupin et al. (21), who conducted a particle image velocimetry (PIV) experiment for an idealized sidewall-type saccular aneurysm. We used the inlet and outlet boundary conditions measured in their experiment in our CFD validation to ensure that our results were comparable to their results. Meshing and CFD analyses were conducted using ANSYS Workbench Fluent (version 19.2; ANSYS Inc., Canonsburg, Pennsylvania, USA). An element of size 0.2 mm was used for the validation model, and the density and viscosity of the fluid were set to 1,200 kg/m^3^ and 0.0038 Pa·s, respectively. The inlet boundary condition was constructed using the Womersley profile, and the pressure profile was applied to the outlet boundary condition. The velocity and streamline were extracted after three cardiac cycles to compare the results of the PIV experiment with the CFD results. The velocity contour and streamline calculated via CFD analysis were consistent with the results of the PIV experiment (Figure 1B).

### CFD Analysis With Stent

Three-dimensional models of the aneurysm and stents were constructed using CATIA computer-aided design software (V5-6R2012; Dassault Systèmes, Paris, France). A stent was constructed only at the aneurysm neck to improve the efficiency of the CFD analysis (23). An element of size 0.2 mm was used for the aneurysm, and an element of size 0.005 mm was generated near the location where the stent was deployed. Overall, 30–50 million elements were used in the CFD analysis. The blood was assumed to be an incompressible Newtonian fluid (24) with the density and viscosity of 1,055 kg/m^3^ and 0.004 Pa·s, respectively (25). The pulsatile flow of the internal carotid artery with a Womersley profile was used as the inlet boundary condition, and zero pressure was used as the outlet boundary condition (26). The blood vessel was assumed to have a rigid wall under non-slip conditions. All the hemodynamic parameters were calculated as systolic after three cardiac cycles.

To evaluate the results of the CFD analysis quantitatively, we compared the following hemodynamic parameters: inflow rate, average velocity, and energy loss (EL). The average velocity and inflow rate into the aneurysm were calculated at plane C, which is located near the aneurysm neck (Figure 1C). The velocity and pressure in planes A and B were extracted to calculate the EL based on the following equation (27):

$$\begin{array}{l} EL=\;\frac{v_{in}A\cdot \left\{\left(\frac{1}{2}\rho v_{in}^{2}+P_{in}\right)-\left(\frac{1}{2}\rho v_{out}^{2}+P_{out}\right)\right\}}{V_{m}} \end{array}$$

where *V*_*m*_ represents the volume of the model between planes A and B. ρ and A are the density and area at the inlet, respectively. *v*_*in*_ and *P*_*in*_ represent the average velocity and pressure, respectively, at the inlet (plane A), and *v*_*out*_ and *P*_*out*_ represent the average velocity and pressure, respectively, at the outlet (plane B). The EL indicates the amount of blood flowing into the aneurysm. We calculated the reduction rate of the EL to indicate the effect of stenting compared with the unstented case. Therefore, a higher EL reduction rate indicates less blood flow into the aneurysm.

## Results

### Comparison of the Flow Diversion Effect and MCR

**Figure 2:**
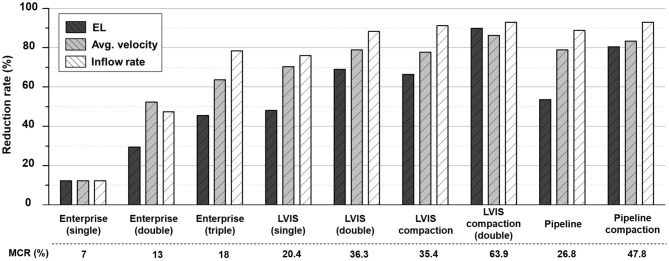
Comparison of the MCR and reduction rates for the hemodynamic parameters according to the overlapping and compaction of Enterprise, LVIS, and Pipeline devices. MCR, metal coverage rate.

The changes in the MCR and hemodynamic parameters caused by overlapping and compacting the stents are summarized in Table 1. As the MCR was increased by overlapping and compaction, the reduction rate of the hemodynamic parameters increased accordingly, and the correlation was statistically significant (Figure 2).

**Table 1:** Summary of changes in the MCR and hemodynamic parameters caused by overlapping or compacting the stents used in this study.

**Device**	**MCR** **(%)**	**EL (W/m^**3**^)** **(reduction rate %)**	**Avg. velocity (m/s)** **(reduction rate %)**	**Inflow rate (mm^**3**^/s)** **(reduction rate %)**
Control	0.0	66.09 (0.00)	0.0114 (0.00)	224.4 (0.00)
Enterprise (single)	7.0	58.01 (12.23)	0.01 (12.28)	196.4 (12.33)
Enterprise (double)	13.0	46.7 (29.34)	0.00543 (52.37)	119.0 (47.36)
Enterprise (triple)	18.0	36.05 (45.45)	0.00415 (63.60)	48.5 (78.43)
LVIS (single)	20.4	34.38 (47.98)	0.00339 (70.26)	54.0 (75.89)
LVIS (double)	36.3	20.49 (69.00)	0.00241 (78.86)	26.5 (88.24)
LVIS compaction (single)	35.4	22.21 (66.39)	0.00254 (77.72)	20.1 (91.15)
LVIS compaction (double)	63.9	6.72 (89.83)	0.00156 (86.32)	15.9 (92.93)
Pipeline	26.8	30.68 (53.58)	0.00241 (78.86)	24.9 (88.82)
Pipeline compaction	47.8	12.9 (80.48)	0.0019 (83.33)	15.7 (92.90)
Pearson correlation^*^ (coefficient, *p*-value)		−0.961 ( ≤ 0.001)	−0.82 (0.004)	−0.805 ( ≤ 0.001)

* *Correlation between the actual value of each hemodynamic parameter and the MCR*.

*MCR, metal coverage rate; EL, energy loss*.

### Comparison of the Flow Diversion Effects of Single Stents

**Figure 3:**
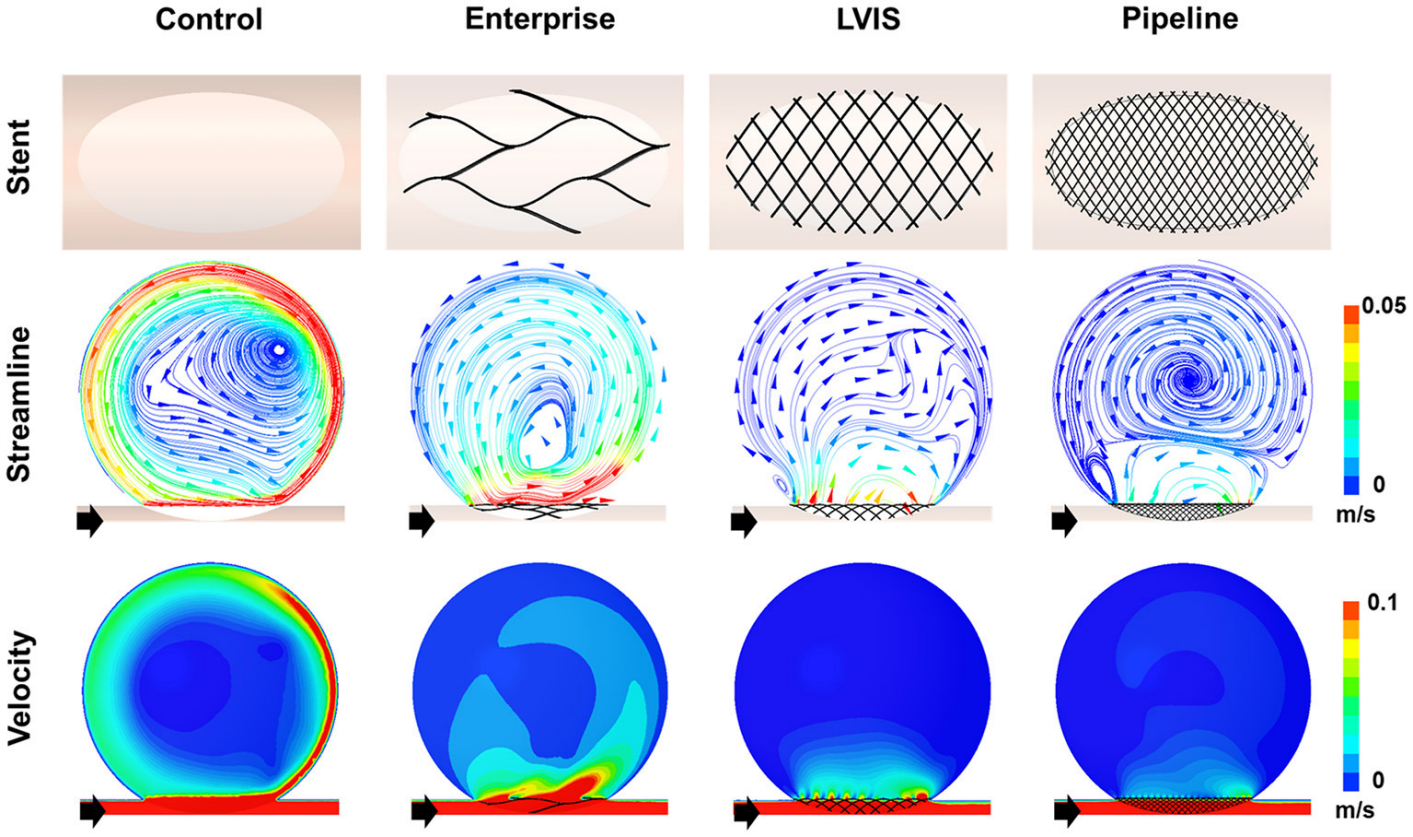
Visualization of the hemodynamic modification of the idealized aneurysm model by each stent. The stent configurations in the idealized aneurysm model and the streamlines and velocity contours calculated using CFD analysis are displayed. The black arrows indicate the flow direction. With the Enterprise stents, the velocity magnitude of the jet flow was decreased compared with that of the control, but the flow pattern of inflow from the distal part of the aneurysmal neck and outflow to the proximal side did not change. In contrast, the LVIS and Pipeline devices disrupted and changed the direction of the inflow jet. Particularly with the Pipeline device, the jet flow was not transferred into the aneurysmal dome due to the remarkable reduction of inflow jet. This led to a silent vortex in the aneurysmal sac due to a separation in the hemodynamics of the aneurysmal dome and neck. CFD, computational fluid dynamics.

First, we compared the hemodynamic modifications induced by each stent (Figure 3). An intra-aneurysmal flow diversion was observed with all the three stents compared with the unstented ideal aneurysm model used as the control. However, the changes in flow pattern and velocity magnitude differed according to the MCR. As the MCR of the different stents increased in the order of Enterprise, LVIS, and Pipeline, the velocity magnitude showed a tendency to decrease. With the Enterprise stent, the velocity magnitude of the jet flow decreased compared with that of the control, but the flow pattern (inflow from the distal part of the aneurysmal neck and outflow to the proximal) did not change. In contrast, both the LVIS and Pipeline devices disrupted and changed the direction of the inflow jet. In particular, the Pipeline device did not transfer the jet flow into the aneurysmal dome because of its remarkable reduction of the inflow jet. This led to a silent vortex in the aneurysmal sac due to a separation in the hemodynamics of the aneurysmal dome and neck.

The Pipeline device (MCR 26.8%, EL 53.58%, average velocity 78.86%, inflow rate 88.82%) showed the most pronounced reduction rate for all the three parameters. The LVIS stent (MCR 20.4%, EL 47.98%, average velocity 70.26%, inflow rate 75.89%) showed a higher reduction rate for all the three parameters than the Enterprise stent (MCR 7.0%, EL 12.23%, average velocity 12.28%, inflow rate 12.33%) (Table 1; Figure 2).

### Comparison of Stent Overlapping Effects

**Figure 4:**
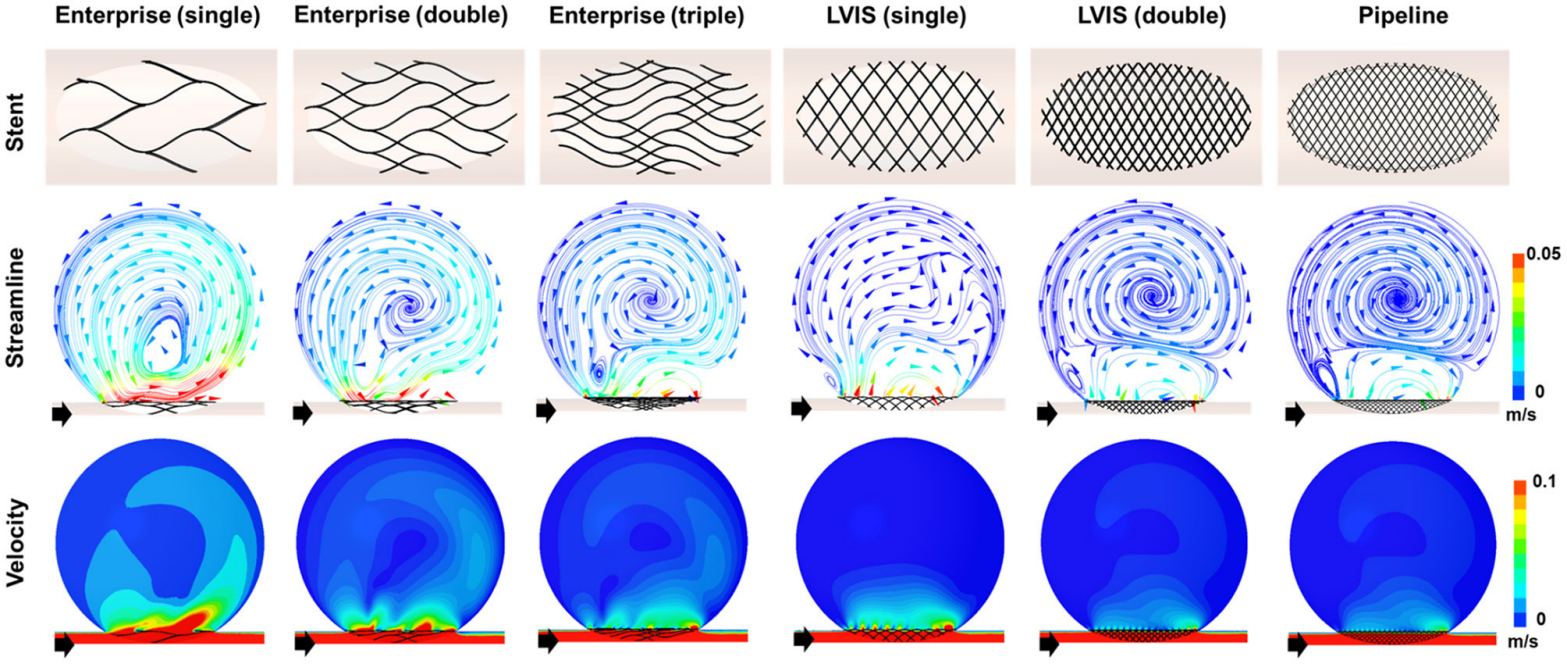
Visualization of the hemodynamic modifications induced by overlapping the stents. The stent configurations deployed in the idealized aneurysm model and the streamlines and velocity contours calculated using CFD analysis are displayed. The black arrows indicate the flow direction. The overlapped Enterprise stents decreased the volume of the inflow jet by disrupting the jet flow, but they did not change the direction of the intra-aneurysmal jet flow even when three stents were used. In contrast, the single LVIS stent without overlapping changed the direction of the inflow jet. A separation of the hemodynamics of the aneurysmal dome and neck, similar to that observed with the Pipeline device, was observed when the two LVIS stents were overlapped. CFD, computational fluid dynamics.

The results of the CFD analysis for stent overlapping are shown in Figure 4. Simulations were performed to overlap one, two, and three Enterprise stents and one and two LVIS stents, using the Pipeline device as the control, to confirm the overlapping effects of the Enterprise and LVIS stents. The changes in the velocity magnitude with stent overlapping tended to follow the change in the MCR. However, the change in the flow pattern according to the stent overlap differed for each stent. With the overlapped Enterprise devices, the volume of the inflow jet decreased due to the disruption of the jet flow. However, the direction of the intra-aneurysmal jet flow did not change even when three Enterprise stents were used together. In contrast, a change in the direction of the inflow jet was observed with a single LVIS stent without overlapping. Moreover, when two LVIS stents were overlapped, the separation of the hemodynamics of the aneurysmal dome and neck was similar to that observed with a single Pipeline device.

In terms of parameter reduction, a single LVIS stent (MCR 20.4%, EL 47.98%, average velocity 70.26%, inflow rate 75.89%) induced a reduction in all the hemodynamic parameters comparable to the effect of three overlapped Enterprise stents (MCR 18.0%, EL 45.45%, average velocity 63.60%, inflow rate 78.43%). Two uncompacted LVIS stents showed a better flow diversion effect (MCR 36.3%, EL 69.00%, average velocity 78.86%, inflow rate 88.24%) than a single LVIS stent. Moreover, the effect of two uncompacted LVIS stents was similar to that of a single uncompacted Pipeline device (MCR 26.8%, EL 53.58%, average velocity 78.86%, inflow rate 88.82%).

### Comparison of Stent Compacting Effects

**Figure 5:**
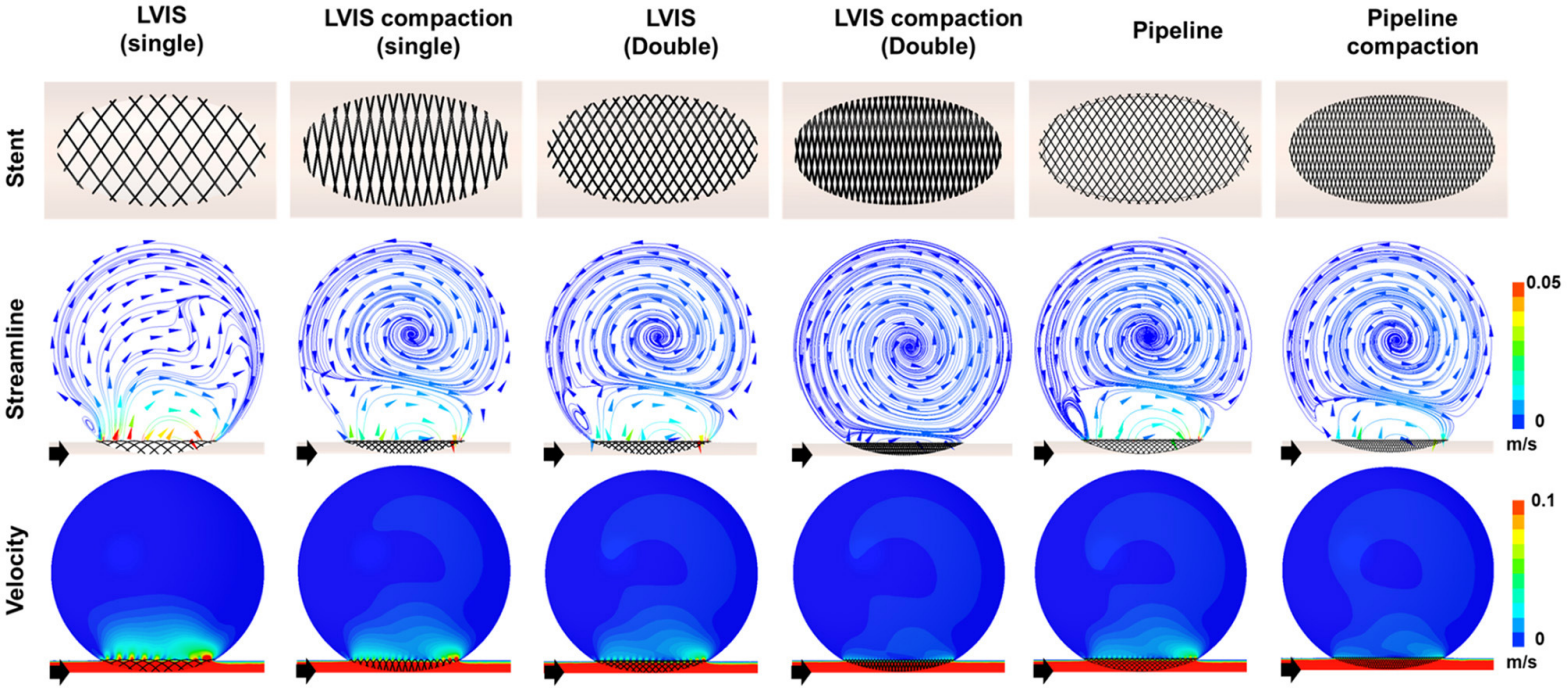
Visualization of the hemodynamic modification induced by compacting the stents. The stent configuration deployed in an idealized aneurysm model and the streamlines and velocity contours calculated using CFD analysis are displayed. The black arrows indicate the flow direction. Stent compaction improved the flow diversion effect of both the LVIS and Pipeline devices. The compacted LVIS stent induced a similar decrease in the velocity magnitude and change in the flow pattern as the two uncompacted LVIS devices or single uncompacted Pipeline device. CFD, computational fluid dynamics.

To demonstrate the effect of stent compaction, we performed simulations in the following order: a single uncompacted LVIS stent, a single compacted LVIS stent, two uncompacted LVIS stents, two compacted LVIS stents, a single uncompacted Pipeline device, and a single compacted Pipeline device (Figure 5). Compaction induced a better flow diversion effect than the lack of compaction with either device. The single compacted LVIS stent induced a similar decrease in the velocity magnitude and change in the flow pattern as the two uncompacted LVIS stents or single uncompacted Pipeline device. In particular, vortex formation within the aneurysm and the separation of the hemodynamics of the aneurysmal dome and neck were observed with the single compacted LVIS stent. The flow diversion effect of the Pipeline device was also improved by compaction, which reduced the size of the inflow jet compared with that of the uncompacted Pipeline device. The two compacted LVIS stents eliminated most of the jet flow and almost completely separated the flow inside the aneurysm from the flow near the aneurysm neck.

The single compacted LVIS stent (MCR 35.4%, EL 66.39%, average velocity 77.72%, inflow rate 91.15%) induced a reduction in all the hemodynamic parameters comparable to the effect of the two uncompacted LVIS stents (MCR 36.3%, EL 69.00%, average velocity 78.86%, inflow rate 88.24%) or the single uncompacted Pipeline device (MCR 26.8%, EL 53.58%, average velocity 78.86%, inflow rate 88.82%). Similarly, the two compacted LVIS stents (MCR 63.9%, EL 89.83%, average velocity 86.32%, inflow rate 92.93%) showed a flow diversion performance comparable to that of the single compacted Pipeline device (MCR 47.8%, EL 80.48%, average velocity 83.33%, inflow rate 92.90%).

## Discussion

The purpose of this study was to analyze quantitatively the flow diversion effects of stents with different MCRs and the effects of stent overlapping and compaction. We observed that (1) a single LVIS stent showed a comparable flow diversion effect to three overlapping Enterprise stents, (2) two-overlapped, uncompacted LVIS stents had a similar flow diversion performance to a single uncompacted Pipeline device, and (3) a single compacted LVIS stent and two-overlapped, uncompacted LVIS stents produced a similar performance. These findings support the following conclusions: (1) A stent with a high MCR can reduce intra-aneurysmal flow activity better than a stent with a low MCR. (2) Increasing the MCR through stent overlapping can induce a flow diversion effect as large as that induced by a flow diverter. (3) It is important to increase the MCR through stent compaction to induce a sufficient flow diversion effect. The proper compaction of a braided stent induces a better flow diversion effect compared with that induced by multiple overlapped, uncompacted stents.

Intra-aneurysmal hemodynamics plays an important role in aneurysmal growth and rupture (28). Previous studies have reported that the flow diversion effect induced by a stent can alter intra-aneurysmal hemodynamics and that the MCR is an important parameter for determining the flow diversion effect induced by a stent (12, 29). As shown in Figure 3, the Enterprise, LVIS, and Pipeline devices all exhibited a flow diversion effect compared with the unstented control case. The Pipeline and Enterprise devices had the highest and lowest flow diversion effects, respectively, which were consistent with their MCRs. Dholakia et al. (30) compared the flow diversion effects of five neurovascular stents using contrast concentration–time curves within the aneurysm. They reported that the LVIS stents showed better flow diversion effects than the Enterprise stents, which is consistent with the results of this study. Jankowitz et al. (31) studied the flow diversion effects of two low-metal-coverage stents (Neuroform Atlas and Enterprise), the LVIS blue stent, and the Pipeline device and observed trends similar to our results. These findings indicate that the MCR of a stent is associated with flow diversion.

As the MCR of a stent is determined by its mechanical properties, such as its number, thickness, and the weave angle of the stent wire (5, 32), each commercialized stent has a constant MCR and thus produces a constant flow diversion effect. Although each stent has a unique MCR, the MCR can be increased by overlapping multiple stents. Tremmel et al. (33) used CFD to study the hemodynamic changes induced by overlapping Enterprise stents and reported that overlapping two or three Enterprise stents sequentially decreased hemodynamic parameters, such as wall shear stress, velocity, turnover time, and pressure. Kojima et al. (34) studied the flow diversion effects of implanting multiple Enterprise stents. They reported that two Enterprise stents yielded a greater reduction in the intra-aneurysmal pressure and wall shear stress compared with a single Enterprise stent, but the reduction in velocity did not differ significantly. Furthermore, the flow diversion effect of two Enterprise stents in Kojima's study was not as large as that of a single Pipeline device. On the other hand, Roszelle et al. (13) conducted a PIV experiment and reported that overlapping three Enterprise stents produced a flow diversion effect similar to that of a Pipeline device. All the three studies on the effects of overlapping Enterprise stents confirmed that overlapping correlated with an increase in the flow diversion effect. However, the flow diversion effect of Enterprise stents varied among the studies, possibly because of the differences in study designs, such as the geometry and size of the aneurysm and stent, and the validation tools and hemodynamic parameters used. In this study, we set an ideal sidewall aneurysm formed on a straight parent artery to control for factors other than the MCR of the stent that affect the flow diversion effect. As we overlapped one to three Enterprise stents, the MCR increased from 7 to 13 and 18%, and the reduction rate of the velocity, EL, and inflow increased sequentially. However, the MCR of the three overlapped Enterprise stents were still inferior to those of a single uncompacted LVIS stent and the flow diversion effect of three overlapped Enterprise was not better than a single uncompacted LVIS stent (Figure 5). Therefore, when the MCR of a single stent is low, overlapping multiple stents results in a limited increase in the MCR. Therefore, clinicians using a stent to create flow diversion for the treatment of aneurysms must consider the MCR of the stent.

In this study, we investigated the effects of overlapping LVIS stents. Overlapping two uncompacted LVIS stents induced a flow diversion effect as large as that induced by a single Pipeline device. Wang et al. (14) also used CFD to compare the flow diversion effects of LVIS, Enterprise, and Pipeline devices. They reported that two LVIS stents can induce a greater flow diversion effect than a single Pipeline device, which is consistent with our results. The LVIS is a braided stent made by braiding a single nitinol wire. Braided stents are characterized by the ability to rearrange the filament to adapt to vascular geometry, which induces various MCRs. The MCR of an uncompacted deployed LVIS is 11–12% (8). However, an MCR more than 20% is possible, depending on the size discrepancy between the parent artery and the stent (35). In this study, the MCR of the 3.5 mm LVIS stent installed in the 4 mm parent artery was 20.4%, and the MCR of the two overlapped LVIS stents was 36.3%. Therefore, the overlapping effect of LVIS stents can yield hemodynamic advantages in real-world practice.

Compaction can also increase the MCR. A braided stent can generate various mesh densities as the wires of the stent are rearranged, making it possible to increase the MCR during stent deployment by using the push-pull technique (19). Previous studies have shown that increasing the MCR of Pipeline devices through compaction improves their flow diversion effect (9, 17). Furthermore, Tian et al. (36) reported that compacted LVIS stents could induce a flow diversion effect comparable to that induced by uncompacted Pipeline devices. We also observed that stent compaction affects the flow diversion effect. As shown in Figure 5, compaction increased the MCR of both the LVIS and Pipeline devices, which improved the flow diversion effect. We also observed that compacting a single LVIS stent induced a flow diversion effect as large as that induced by two overlapping LVIS stents or a single, uncompacted Pipeline device. Moreover, overlapping two compacted LVIS stents induced a flow diversion effect as large as that induced by a single compacted Pipeline device. The results of this study on stent compaction may differ from those in real-world practice because our results are derived from an assumed ideal condition to maximize the MCR. However, as all the stent experiments in this study were conducted under the same conditions, our results showing the relative flow diversion effects of the stents with and without overlapping or compaction may still be informative for actual clinical practice.

This study has some limitations. First, as explained, our experiments were conducted under the assumption of an ideal condition; therefore, the results obtained in real practice may be different. We assumed an idealized sidewall-type saccular aneurysm to exclude factors other than the stent properties that affect the flow diversion effect. However, the flow diversion effects of the stents could vary in real practice depending on factors, such as the shape of the aneurysm, the geometry of the parent artery, and the degree of wall apposition between the stent and the parent artery (37–39). Moreover, our hemodynamic study was performed under the assumption that the virtual stent covered the entire aneurysm neck uniformly with a maximum MCR. When multiple stents are overlapped in a clinical setting, they cannot be placed such that they divide the stent cells equally, as assumed in the CFD simulations. In addition, when a braided stent is compacted, the metal coverage on the aneurysm neck can vary along different segments, even on a single device (16). Second, our CFD analysis has technical limitations. Several assumptions for the CFD analysis, such as the properties of blood and the boundary conditions, were set for the generalized conditions of intracranial circulation; however, they might not reflect all patient-specific conditions. Therefore, the flow diversion effects of the stents in real-world practice may differ from the results presented here. Nonetheless, this proof-of-concept study demonstrates the maximum capacity of the flow diversion effects of the stents, including the effects of overlapping and compaction. To prove the effects of overlapping and compaction, we needed to control for other conditions affecting the flow diversion effect. Although our results may differ somewhat from the actual flow diversion effects of the stents, our objective comparison of the changes in aneurysm hemodynamics induced by overlapping and compaction extends the current understanding of how the flow diversion effect depends on the type of stent, overlapping, and compaction.

## Conclusions

We observed that a single LVIS stent exhibited a flow diversion effect similar to that of three overlapped Enterprise stents. Compacting a single LVIS stent was as effective in terms of flow diversion as overlapping two LVIS stents, and similar results were confirmed for the Pipeline device. The MCR of a stent correlates with its flow diversion effect. Overlapping and compcation can increase the MCR of an intracranial stent and improve the flow diversion effect to match that of a flow diverter.

## Data Availability Statement

The original contributions generated for the study are included in the article/supplementary material, further inquiries can be directed to the corresponding author/s.

## Author Contributions

SK and HY gathered the data and drafted the manuscript in collaboration. IH assisted in the discussions and reviewed the manuscript. JO and YK conceptualized the study and supervised the process, corresponding to each field of specialty. All authors approved the final version of the manuscript.

## Conflict of Interest

The authors declare that the research was conducted in the absence of any commercial or financial relationships that could be construed as a potential conflict of interest.

## Publisher's Note

All claims expressed in this article are solely those of the authors and do not necessarily represent those of their affiliated organizations, or those of the publisher, the editors and the reviewers. Any product that may be evaluated in this article, or claim that may be made by its manufacturer, is not guaranteed or endorsed by the publisher.
